# Pharmacodynamic and Pharmacokinetic Drug Interactions between Fexuprazan, a Novel Potassium-Competitive Inhibitor, and Aspirin, in Healthy Subjects

**DOI:** 10.3390/pharmaceutics15020549

**Published:** 2023-02-07

**Authors:** JungJin Oh, Eunsol Yang, In-Jin Jang, Hyejung Lee, Hokyun Yoo, Jae-Yong Chung, SeungHwan Lee, Jaeseong Oh

**Affiliations:** 1 Department of Clinical Pharmacology and Therapeutics, Seoul National University College of Medicine and Hospital, Seoul 03080, Republic of Korea; 2 Medical Research Center, Seoul National University, Seoul 03080, Republic of Korea; 3 Daewoong Pharmaceutical Co., Ltd., Seoul 06170, Republic of Korea; 4 Department of Clinical Pharmacology and Therapeutics, Seoul National University Bundang Hospital, Seongnam 13620, Republic of Korea

**Keywords:** drug interactions, aspirin, fexuprazan, potassium-competitive acid blocker, gastrointestinal complications

## Abstract

Acid-reducing agents are commonly used for the prevention of aspirin-induced gastrointestinal complications such as peptic ulcers. As a novel potassium-competitive acid blocker, fexuprazan is expected to prevent aspirin-induced gastrointestinal complications. This randomized, open-label study aimed to evaluate the pharmacodynamic and pharmacokinetic interactions between aspirin and fexuprazan in healthy Koreans. Subjects randomized to the aspirin group received 500 mg aspirin in combination with 80 mg fexuprazan. For the fexuprazan group, fexuprazan 80 mg was administered alone and then in combination with aspirin 500 mg. Platelet aggregation inhibited by aspirin and the pharmacokinetic parameters of aspirin and fexuprazan were compared between monotherapy and combination therapy. A total of 22 subjects completed the study. The platelet aggregation-inhibitory activity and systemic exposure to aspirin were not significantly affected by fexuprazan coadministration. The systemic exposure of fexuprazan was decreased up to 20% by aspirin coadministration, which was not regarded as clinically meaningful considering the previously reported exposure–response relationship. In conclusion, there were no clinically relevant pharmacodynamic or pharmacokinetic interactions between aspirin and fexuprazan. This finding suggests the potential of fexuprazan for the prevention of aspirin-induced gastrointestinal complications, serving as a baseline for optimizing its therapeutic application with aspirin.

## 1. Introduction

Aspirin, an antiplatelet agent, has been widely used for primary and secondary prevention of cerebrovascular, cardiovascular, and peripheral arterial diseases [1]. Low-dose aspirin (LDA) at 75–325 mg effectively prevents occlusive vascular events by up to 25% in high-risk patients with myocardial infarction and stroke [2]. The action of aspirin is dose-dependent; LDA inhibits thromboxane A2 through the irreversible inhibition of cyclooxygenase-1 (COX-1), exhibiting anti-platelet effects, whereas a high dose of aspirin leads to anti-inflammatory and anti-pyretic effects through the dual inhibition of COX-1 and COX-2 enzymes [3].

It has also been reported that the long-term use of aspirin poses the risk of developing gastrointestinal bleeding and gastric/duodenal ulcers, even at low doses [4,5]. Hence, combination therapies with gastrointestinal protective agents are recommended [6], the effectiveness of which was well appreciated in a previous study showing that esomeprazole and lansoprazole had preventative effects on gastrointestinal complications and symptoms [7,8]. Furthermore, combination therapy with proton pump inhibitors (PPIs) reduced the risk of hospitalization from major gastrointestinal complications [7].

Despite the potentially increased risk of cardiovascular adverse events (AEs) by the drug‒drug interactions (DDIs) between aspirin and PPIs, it was demonstrated that the simultaneous use of PPIs and aspirin caused neither significant pharmacokinetic (PK) interactions [9] nor pharmacodynamic (PD) interactions associated with platelet aggregation and other adverse effects [10,11,12].

Fexuprazan is a novel potassium-competitive acid blocker (P-CAB) that was developed to overcome the limitations of PPIs. In contrast to PPIs, fexuprazan remains effective for a long time with minimal effects from CYP2C19 polymorphisms. Moreover, it can be activated even under nonacidic conditions, and it has a faster onset of action [13]. Meanwhile, fexuprazan is mainly metabolized by CYP3A4 [14], which might result in CYP3A4-mediated DDIs.

Although fexuprazan can be used in combination with aspirin as an alternative to PPIs, the PK and PD interactions between aspirin and fexuprazan have never been evaluated in a quantitative fashion. Therefore, the present study aimed to examine the PK and PD interactions between aspirin and fexuprazan.

## 2. Materials and Methods

The study protocol and the informed consent form were approved by the Ministry of Food and Drug Safety and the Institutional Review Board of Seoul National University. This study was performed in accordance with the ethical principles of the Declaration of Helsinki, the Korean Good Clinical Practice, and relevant regulatory requirements (NCT05304845). Written informed consent was obtained from all subjects before performing any study-related procedures.

### 2.1. Study Design

This was a randomized, open-label, two-period, fixed-sequence crossover study (Figure S1). Based on the exploratory and descriptive characteristics of the study aim, the calculation of study power was not considered to determine the sample size. The enrolled subjects were randomly assigned to the aspirin group or the fexuprazan group at a ratio of 1:1. For the aspirin group, a single oral dose of aspirin 500 mg was administered alone in the first period and then in combination with fexuprazan 80 mg after 4 days of pretreatment with once-daily doses of fexuprazan 80 mg in the second period, and there was a 10-day washout period between each period. For the fexuprazan group, once-daily doses of fexuprazan 80 mg were administered alone for 5 days in the first period and then in combination with aspirin 500 mg for 5 days in the second period. The subjects received their respective treatment with 150 mL of water after overnight fasting.

For the aspirin group, serial blood samples were collected at predose, 1.5, 4, 6, 8, 10, 12, 24, and 48 h after aspirin dosing for the PD analysis and at predose, 0.17, 0.33, 0.5, 1, 1.5, 2, 3, 4, 6, 8, 12, 24, 36, and 48 h after aspirin dosing for the PK analysis. For the PD evaluation of aspirin, 16.2 mL of blood was taken in a citrate tube and then analyzed by both arachidonic acid-induced and collagen-induced platelet aggregation assays. For the PK evaluation of aspirin and its metabolite, salicylic acid, 10 mL of blood was taken in a sodium fluoride and potassium oxalate tube at each sampling point and subsequently centrifuged at 3000 rpm for 10 min at 4 °C, and 1.0 mL of supernatant was transferred to Eppendorf tubes and stored at −70 °C until analysis. For the fexuprazan group, serial blood samples for PK analysis were collected at predose, 0.5, 1, 1.5, 2, 2.5, 3, 4, 6, 8, 12, 24, 36, and 48 h after the 5th dose of fexuprazan (i.e., the last dose) and at predose before the 3rd and 4th doses of fexuprazan. For PK evaluation of fexuprazan and its metabolite, M14, 6 mL of blood was taken in a sodium heparin tube for each sampling point and subsequently centrifuged at 3000 rpm for 10 min at 4 °C, and 1.0 mL of supernatant was transferred to Eppendorf tubes and stored at −70 °C until analysis.

### 2.2. Study Population

Healthy Koreans who were 19 to 50 years old with a body weight of 50.0 to 90.0 kg and a body mass index of 18.0 to 27.0 kg/m^2^ were eligible for this study. The major exclusion criteria were as follows: platelet count < 150,000/μL, prothrombin time international normalized ratio (PT INR) > 1.2, activated partial thromboplastin time (aPTT) > 40 s, Modification of Diet in Renal Disease (MDRD)-estimated glomerular filtration rate (eGFR) < 60 mL/min/1.73 m^2^, and any hypersensitivity to drugs, including P-CABs and aspirin.

### 2.3. Platelet Aggregation Assays

Whole blood was collected in a BD Vacutainer tube (Becton Dickinson, NJ, USA) containing 3.8% sodium citrate and used within one hour. The blood was centrifuged at 950 rpm for 10 min to recover platelet-rich plasma (PRP). The remaining plasma was recentrifuged at 3000 rpm for 10 min to recover platelet-poor plasma (PPP). Both PRP and PPP were prepared at room temperature. PPP was used to adjust the platelet number in the PRP to 200,000–400,000/mm^3^ and as a baseline reference. Platelet aggregation was triggered by 0.5 mmol/L arachidonic acid and 2 μg/mL collagen. The platelet aggregation was measured with a Chronolog Lumi-Aggregometer (Model 700, Chrono-Log, PA, USA).

### 2.4. Determination of the Drug Concentrations

Plasma concentrations of aspirin, fexuprazan, and M14 were determined by liquid chromatography–tandem mass spectrometry (LC‒MS) using an AB SCIEX API 5000 mass spectrometer (SCIEX, Foster City, CA, USA). The salicylic acid plasma concentration was determined by LC‒MS using an AB SCIEX API 4000 mass spectrometer (SCIEX). For the aspirin specimens, 55 μL of plasma was mixed with 10 μL of the internal standard (IS) solution (i.e., aspirin-d_4_ (Toronto Research Chemical, Toronto, Canada)) and 200 μL of acetonitrile. The mixed solutions were vortexed and centrifuged. Then, 150 μL of supernatant was dried with nitrogen gas and redissolved by adding 100 μL of 0.1% formic acid in 0.1% acetonitrile. Finally, the supernatant was subjected to the LC‒MS system for analysis. Chromatographic retention was done on ACE 3 C18, 2.1 × 30 mm, 3 μm column (Aberdeen, Scotland) at a flow rate of 0.4 mL/min. Multiple reaction monitoring (MRM) of aspirin and IS solution were 179.0 > 137.1 *m*/*z* and 183.0 > 141.0 *m*/*z*, respectively. For salicylic acid specimens, 55 μL of plasma was mixed with 20 μL of IS solution (i.e., salicylic acid-d_4_ (Toronto Research Chemical, Toronto, ON, Canada)) and 800 μL of acetonitrile. The mixed solutions were centrifuged, and the supernatant was subjected to LC‒MS analysis. Chromatographic retention was performed on ACE 3 C18, 2.1 × 50 mm, 3 μm column (Aberdeen, Scotland) at a flow rate of 0.4 mL/min. MRM of salicylic acid and IS solution were 136.9 > 93.0 *m*/*z* and 140.9 > 97.0 *m*/*z*, respectively. For the fexuprazan and M14 plasma specimens, 50 μL of plasma was mixed with 50 μL of IS solution (i.e., a 2-in-1 solution of [CD_3_]fexuprazan and M14 deuterium (Daewoong Pharmaceutical Co., Ltd., Seoul, Republic of Korea)) and 450 μL of acetonitrile. The mixed solutions were centrifuged, and the supernatant was subjected to LC‒MS analysis. Chromatographic separation was performed on ACE 3 C18, 2.1 × 30 mm, 3 μm column (Aberdeen, Scotland) at a flow rate of 0.5 mL/min. MRM of fexuprazan, [CD_3_]fexuprazan, M14 and M14 deuterium were 411.3 > 380.0 *m*/*z*, 414.3 > 380.2 *m*/*z*, 412.3 > 235.1 *m*/*z*, and 415.3 > 238.1 *m*/*z*, respectively.

The quantification limits of the aspirin, salicylic acid, fexuprazan, and M14 in the plasma samples were 5 μg/L, 100 μg/L, 1.10 μg/L and 10.2 μg/L, respectively. The accuracies for aspirin, salicylic acid, fexuprazan, and M14 in the plasma samples were 90.6–100.0%, 98.4–103.0%, 101.5–104.0%, and 94.2–98.7%, respectively. The precision coefficients of variation for aspirin, salicylic acid, fexuprazan, and M14 in the plasma samples were ≤3.6%, ≤1.9%, ≤4.0%, and ≤4.4%, respectively.

### 2.5. Pharmacodynamic and Pharmacokinetic Analyses

The following PD and PK parameters were derived from the noncompartmental method using Phoenix^®^ WinNonlin^®^ version 8.2 (Certara, St. Louis, MO, USA). The degree of platelet aggregation maximally inhibited by aspirin (i.e., maximum platelet aggregation (%)) was defined as the value of the lowest platelet aggregation (%) in each individual subject and determined from the observed platelet aggregation-time profiles. The area under the concentration-time curve (AUC) from time 0 to the time of the last quantifiable concentration (AUC_last_) or the AUC for a dosing interval at steady state (AUC_τ,ss_) was calculated by the linear-up and log-down trapezoidal rule, and the AUC from 0 to infinity (AUC_inf_) was calculated as the sum of AUC_last_ and the last measurable concentration divided by the terminal elimination rate constant (λ_z_). The maximum plasma concentration (C_max_) or C_max_ at steady state (C_max,ss_) and the time to reach C_max_ or C_max,ss_ (T_max_ or T_max,ss_) were obtained directly from the observed plasma concentration-time profiles. The terminal elimination half-life (t_1/2_) or the t_1/2_ at steady state (t_1/2,ss_) was calculated as ln (2) divided by λ_z_. The metabolic ratio was calculated as the AUC_last_ or AUC_τ,ss_ ratio of the respective metabolite to the parent molecule.

### 2.6. Safety Assessments

Safety was assessed based on AE monitoring, clinical laboratory tests (hematology, chemistry, coagulation, and urinalysis), vital signs, physical examination, and 12-lead electrocardiogram (ECG). All findings from the safety parameters were evaluated by the investigators with regard to their clinical significance and their relationship with the treatment.

### 2.7. Statistical Analysis

Statistical analysis was carried out using SAS^®^ version 9.4 (SAS Institute Inc., Cary, NC, USA). For the aspirin group, the PD and PK parameters of aspirin were summarized by treatment and compared between aspirin monotherapy and combination therapy with fexuprazan. For the fexuprazan group, the PK parameters of fexuprazan were summarized by treatment and compared between fexuprazan monotherapy and combination therapy with aspirin. For each group, analysis of variance was performed for the log-transformed AUC and C_max_, and the effect of the coadministration of aspirin on the PK of fexuprazan or vice versa was evaluated by estimating the geometric mean ratios (GMR) of combination therapy to monotherapy and the corresponding 90% confidence intervals (CIs).

## 3. Results

### 3.1. Study Population

A total of 27 healthy Korean subjects were enrolled and randomized in this study. Among them, 5 subjects discontinued the study due to personal reasons including COVID-19 infection, noncompliance with the study procedure, and serious adverse event of febrile neutropenia (two and three in the aspirin and fexuprazan groups, respectively), and 22 subjects (11 per group) completed the study as planned. PD and PK were analyzed in 22 subjects, including three women, who completed the study and produced sufficient PD and PK data without significant deviations. Safety was assessed in 26 subjects who had received the treatment at least once. The baseline characteristics of the enrolled subjects were similar between the groups. The mean ± standard deviation values for age, height, body weight, and body mass index were 30.59 ± 8.50 years, 172.22 ± 7.74 cm, 69.79 ± 8.95 kg, and 23.49 ± 2.16 kg/m^2^, respectively, and three subjects (11.1%) were women.

### 3.2. Effect of Fexuprazan on the Pharmacodynamics of Aspirin

**Figure 1:**
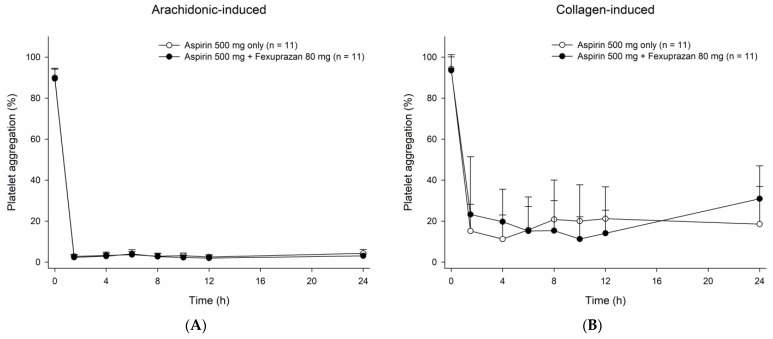
Platelet aggregation-time profiles obtained after a single oral administration of aspirin 500 mg with and without the coadministration of fexuprazan 80 mg in (**A**) arachidonic acid-induced assay and in (**B**) collagen-induced assay. The error bars represent the standard deviations.

**Figure 2:**
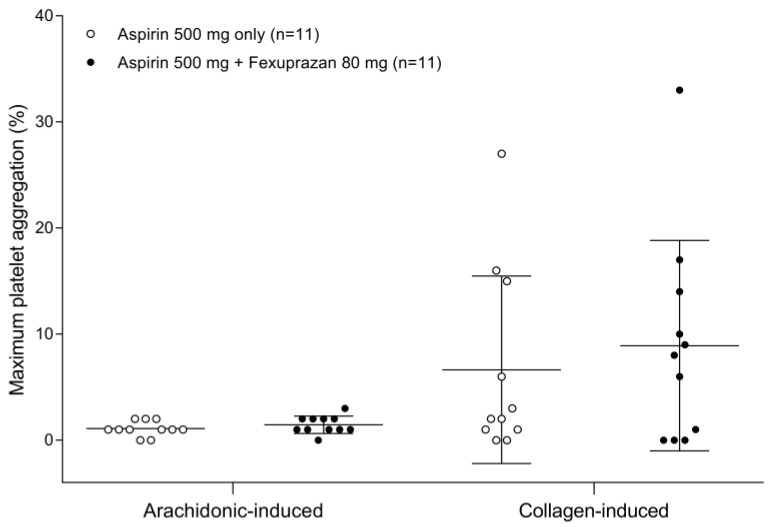
Maximum arachidonic acid-induced and collagen-induced platelet aggregation (%) measured after a single oral administration of aspirin 500 mg alone and coadministered with fexuprazan 80 mg. The horizontal lines, vertical lines, and symbols represent the median, minimum-to-maximum, and individual data, respectively.

The inhibitory activity of aspirin against platelet aggregation appeared not to be significantly affected by the coadministration of fexuprazan. The platelet aggregation-time profiles remained almost intact despite the coadministration of fexuprazan in both arachidonic acid-induced and collagen-induced assays (Figure 1). In the arachidonic acid-induced assay, the mean maximal inhibition of platelet aggregation by aspirin changed negligibly from 1.09% to 1.45% due to the concomitant administration of fexuprazan. Although the inhibition of platelet aggregation exhibited high interindividual variability in the collagen-induced assay, the mean maximal value in the fexuprazan coadministration with aspirin (8.91%) was found to be similar to that (6.64%) of aspirin monotherapy (Figure 2).

### 3.3. Effect of Fexuprazan Coadministration on the Pharmacokinetics of Aspirin

**Figure 3:**
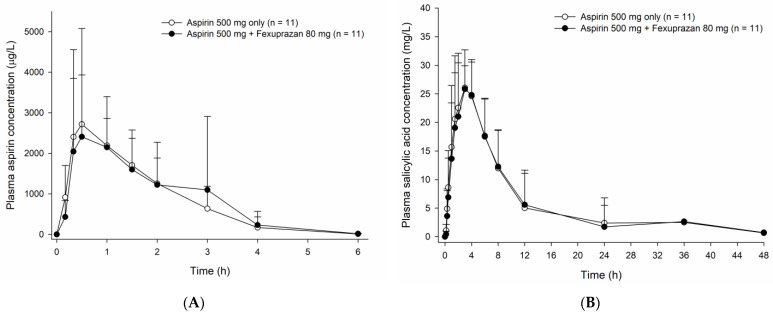
Mean plasma concentration-time profiles of (**A**) aspirin and (**B**) salicylic acid measured after a single oral administration of aspirin 500 mg alone and coadministered with fexuprazan 80 mg. The error bars represent the standard deviations.

The coadministration of fexuprazan had a negligible influence on the PK of aspirin. The plasma concentration-time profiles of aspirin measured upon fexuprazan coadministration were comparable to those obtained in the absence of fexuprazan (Figure 3A). The systemic exposure to aspirin also remained almost unchanged despite fexuprazan coadministration, as confirmed by the similar AUC values in the two cases (Table 1 and Table S1). Furthermore, the PK properties of salicylic acid, an active metabolite of aspirin, appeared to be well conserved irrespective of the concomitant administration of fexuprazan (Figure 3B, Table S2).

### 3.4. Effect of Aspirin on the Pharmacokinetics of Fexuprazan

**Figure 4:**
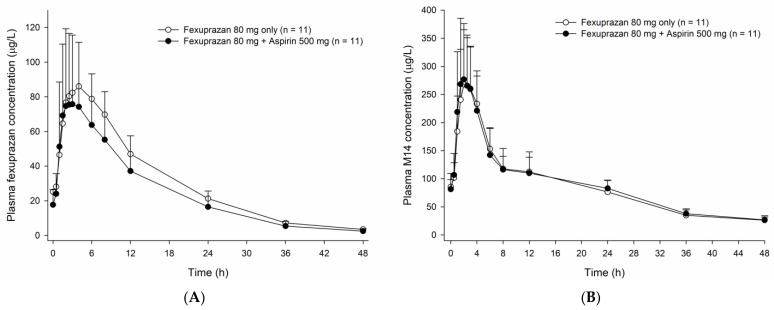
Mean plasma concentration-time profiles of (**A**) fexuprazan and (**B**) M14 measured after multiple oral administrations of fexuprazan 80 mg alone and coadministered with aspirin 500 mg. The error bars represent the standard deviations.

Although the PK of fexuprazan was slightly affected by the coadministration of aspirin, the extent of its influence seemed to be clinically insignificant. The plasma concentration-time profiles of fexuprazan shifted slightly down from fexuprazan monotherapy to the coadministration of aspirin with fexuprazan (Figure 4A). The systemic exposure to fexuprazan decreased by 20% in terms of AUC_τ,ss_ with concomitant administration of aspirin (Table 2 and Table S1). Nonetheless, the PK profile of a major metabolite of fexuprazan (M14) for fexuprazan monotherapy appeared to be similar to that for aspirin coadministration with fexuprazan (Figure 4B, Table S3).

### 3.5. Safety

Throughout the study, a total of 19 treatment-emergent adverse events (TEAEs) were reported in nine subjects (34.6%). In each analysis group, the frequencies of the TEAEs remained similar regardless of coadministration with the counterpart drug. Five cases were reported in two subjects (15.4%) in the fexuprazan coadministration with aspirin group compared to three in two subjects (14.3%) in the aspirin monotherapy group. Five and six TEAEs occurred in three subjects in the aspirin coadministration with fexuprazan group (27.3%) and in the fexuprazan monotherapy group (25.0%), respectively. All TEAEs were mild except for the one serious adverse event of febrile neutropenia, which was observed in a subject who received fexuprazan monotherapy. This event made the subject discontinue the study. The participant was treated with antibiotics and recovered rapidly without any sequelae. Taken together, the coadministration of the counterpart drug caused clinically insignificant changes only, as indicated by clinical laboratory tests, vital signs, 12-lead EGC, and physical examination.

## 4. Discussion

The long-term use of aspirin poses the risk of developing gastrointestinal complications [4,5], which can be prevented by combination therapies with gastrointestinal protective agents [7]. Recently approved fexuprazan can be used in combination with aspirin as an alternative to conventional PPIs. Therefore, the present study investigated DDIs between aspirin and fexuprazan, considering the high potential of fexuprazan in combination therapy with aspirin in clinical settings. The results indicated that the PD and PK interactions between aspirin and fexuprazan were not clinically significant, and that the concomitant administration of these two drugs was well tolerated by healthy subjects. These findings suggest that fexuprazan can be coadministered with aspirin for the purpose of preventing aspirin-induced gastrointestinal complications without an increased risk of cardiovascular and cerebrovascular adverse events.

Standard platelet aggregation assays, light transmittance aggregometry induced by arachidonic acid and collagen, were employed to investigate the effect of fexuprazan coadministration on the PD of aspirin. No clinically meaningful differences in platelet aggregation were observed between aspirin monotherapy and combination therapy with fexuprazan. Both arachidonic acid-induced and collagen-induced platelet aggregation assays revealed clinically meaningful platelet aggregatory inhibition, the minimum of which amounts to 20% and 70% aggregation (i.e., platelet aggregation (%)) in the former and the latter, respectively [15]. As expected, the inhibition of platelet aggregation was maintained for a sufficiently long time, even 24 h after aspirin dosing. Similar to the previously reported results for the vonoprazan-aspirin DDI study [16], the mean value of collagen-induced platelet aggregatory maximum inhibition percent appeared to increase from 6.64 to 8.91% when fexuprazan was coadministered with aspirin. However, this result is considered not clinically meaningful because the collagen-induced assay is highly variable [17], and because sufficient inhibition of platelet aggregation (more than 30% inhibition) was observed by aspirin regardless of the fexuprazan combination. Meanwhile, the arachidonic acid-induced assay exhibited the least variability among the functional platelet aggregability assays, which is not surprising, because it is a substrate of the COX-1 enzyme and is directly related to the mechanism of action of aspirin [17]. This finding suggests that the arachidonic acid-induced platelet aggregation assay may be most appropriate for measuring the inhibition of platelet function by aspirin.

The PK parameters, including AUC, remained almost unchanged during the coadministration of aspirin with fexuprazan, indicating the absence of any meaningful PK DDI between aspirin and fexuprazan. Fexuprazan is mainly metabolized by CYP3A4 [14]. On the other hand, aspirin is rapidly metabolized by aspirin esterase or hydrolyzed automatically into salicylic acid, which is in turn metabolized by CYP450, acyl-CoA N-acyltransferase, and uridine 5′-diphosphoglucronosyltransferases [18]. Consistent with this difference in metabolic mechanisms, no significant DDI was observed between fexuprazan and aspirin in the present clinical study. When coadministered with fexuprazan compared to when administered alone, large variability in plasma aspirin concentration was observed. Considering the absorption of aspirin may be affected by stomach pH [19], an increase in gastric pH by fexuprazan might have caused inter-individual variability during aspirin absorption.

When coadministered with aspirin compared to when administered alone, an approximately 20% decrease in the systemic exposure of fexuprazan was observed in the study. Since fexuprazan has a clear exposure–response relationship explained by the sigmoid E_max_ model [13], it is considered that the corresponding reduction in fexuprazan exposure would result in no clinically meaningful effect on its acid-inhibitory effect.

Low-dose aspirin is usually coadministered with PPIs because aspirin can induce gastrointestinal complications, including gastrointestinal ulcers and bleeding [20]. Although 75–325 mg of low-dose aspirin is used for primary and secondary prevention of cardiovascular and cerebrovascular diseases, 500 mg of aspirin was chosen in this study, because the maximum dose was recommended in the FDA guidelines for conducting DDI studies [21]. A single oral administration was adopted on the grounds that aspirin exhibited a maximum platelet inhibition effect 24 h post dose with a relatively short T_max_ and t_1/2_ of 0.5–1.0 h and 0.4–2.1 h, respectively [22]. Since aspirin blocks the COX-1 enzyme in platelets in an irreversible fashion [23], the lifespan of human platelets was set equal to 10 days for the wash-out period. Fexuprazan was administered once a day to be consistent with the daily dose regimen of aspirin in clinical practice. This administration continued for five days to reach the steady state under consideration of its half-life of 7–9 h [13]. Fexuprazan 80 mg was used as the maximum dose.

It is worth emphasizing that no significant DDI was observed despite the use of the maximum dosage in the FDA guidelines. Hence, there would be no significant DDI between aspirin and fexuprazan during normal clinical applications in which lower doses are used than in this study. As widely adopted in phase I studies, only a small number of empirically selected participants were involved in the present study, and only the short-term effects were addressed. Thus, it should be noted that the long-term PD and PK DDIs associated with safety need to be evaluated in the future with a large population of patients.

## 5. Conclusions

In the present study, there were neither clinically relevant PD nor PK drug interactions between aspirin and fexuprazan. These findings provide persuasive evidence that fexuprazan may serve as an option for the prevention of aspirin-induced gastrointestinal complications, thereby suggesting the clinical usefulness of its concomitant use with aspirin.

## Figures and Tables

**Table 1:** Pharmacokinetic parameters of aspirin determined after a single oral administration of aspirin 500 mg alone and coadministered with fexuprazan 80 mg.

Parameters	Aspirin 500 mg + Fexuprazan 80 mg (N = 11)	Aspirin 500 mg Only (N = 11)	Geometric Mean Ratio ^a^ (90% Confidence Interval)
T_max_ (h)	1.0 [0.3–3.0]	0.5 [0.3–1.5]	NA
AUC_last_ (h·μg/L)	5033.04 ± 979.17	4965.96 ± 1244.60	1.0679 (0.9963–1.1448)
AUC_inf_ (h·μg/L)	5049.74 ± 978.89	4981.70 ± 1248.07	1.0678 (0.9953–1.1457)
C_max_ (μg/L)	4094.55 ± 2179.78	3056.36 ± 1132.98	1.2618 (0.9511–1.6738)
t_1/2_ (h)	0.41 ± 0.05	0.41 ± 0.04	NA

All data are presented as mean ± standard deviation except for T_max_, which is expressed in the form of median (minimum–maximum). Abbreviations: NA, not applicable; T_max_, time to reach maximum plasma concentration; AUC_last_, area under the concentration-time curve (AUC) from time 0 to the time of the last quantifiable concentration; AUC_inf_, AUC from 0 to infinity; C_max_, maximum plasma concentration; t_1/2_, terminal elimination half-life. ^a^ Geometric mean ratio of ‘Aspirin 500 mg + Fexuprazan 80 mg’ to ‘Aspirin 500 mg only’.

**Table 2:** Pharmacokinetic parameters of fexuprazan determined after multiple oral administrations of fexuprazan 80 mg alone and coadministered with aspirin 500 mg.

Parameters	Fexuprazan 80 mg + Aspirin 500 mg (N = 11)	Fexuprazan 80 mg Only (N = 11)	Geometric Mean Ratio ^a^ (90% Confidence Interval)
T_max,ss_ (h)	3.0 [1.5–6.0]	4.0 [2.0–6.0]	NA
AUC_τ,ss_ (h·μg/L)	983.32 ± 507.09	1190.82 ± 511.52	0.7980 (0.7504–0.8486)
C_max,ss_ (μg/L)	85.19 ± 42.44	91.30 ± 37.18	0.8931 (0.8155–0.9780)
t_1/2,ss_ (h)	9.03 ± 1.18	9.06 ± 1.03	NA

All data are presented as mean ± standard deviation except for T_max,ss_, which is expressed in the form of median (minimum–maximum). Abbreviations: NA, not applicable; T_max,ss_, time to reach maximum plasma concentration at steady state; AUC_τ,ss_, area under the concentration-time curve for a dosing interval at steady state; C_max,ss_, maximum plasma concentration at steady state; t_1/2,ss_, terminal elimination half-life at steady state. ^a^ Geometric mean ratio of ‘Fexuprazan 80 mg + Aspirin 500 mg’ to ‘Fexuprazan 80 mg only’.

## Data Availability

The data presented in this study are available upon request from the corresponding author. The data are not publicly available due to privacy or ethical restrictions.

## Supplementary Materials

The following supporting information can be downloaded at: https://www.mdpi.com/article/10.3390/pharmaceutics15020549/s1, Figure S1: Study design; Table S1: Analysis of variance (ANOVA) table for pharmacokinetic parameters of aspirin and fexuprazan; Table S2: Pharmacokinetic parameters of salicylic acid, an active metabolite of aspirin, determined after a single oral administration of aspirin 500 mg alone and coadministered with fexuprazan 80 mg; Table S3: Pharmacokinetic parameters of M14, a metabolite of fexuprazan, determined after multiple oral administrations of fexuprazan 80 mg alone and coadministered with aspirin 500 mg.
