# Supplementary material for: Pharmacodynamic and Pharmacokinetic Drug Interactions between Fexuprazan, a Novel Potassium-Competitive Inhibitor, and Aspirin, in Healthy Subjects
Source: Pharmaceutics. 2023 Feb 7;15(2):549. doi: 10.3390/pharmaceutics15020549 (PMC9958674; doi:10.3390/pharmaceutics15020549)
Supplement: Supplementary file 1 [file pharmaceutics-15-00549-s001.zip › pharmaceutics-2143071-supplementary.pdf]

Supplementary Materials

Pharmacodynamic and Pharmacokinetic Drug Interactions Between Fexuprazan, a Novel Potassium-Competitive Inhibitor, and Aspirin, in Healthy Subjects

JungJin Oh [1,†], Eunsol Yang [1,2,†], In-Jin Jang [1], Hyejung Lee [3], Hokyun Yoo [3], Jae-Yong Chung [4], SeungHwan Lee [1] and Jaeseong Oh [1,*]

[1] Department of Clinical Pharmacology and Therapeutics, Seoul National University College of Medicine and Hospital, Seoul 03080, Republic of Korea
[2] Medical Research Center, Seoul National University, Seoul 03080, Republic of Korea
[3] Daewoong Pharmaceutical Co., Ltd., Seoul 06170, Republic of Korea
[4] Department of Clinical Pharmacology and Therapeutics, Seoul National University Bundang Hospital, Seongnam 13620, Republic of Korea
* Correspondence: johan25@snu.ac.kr
† These authors contributed equally to this work.

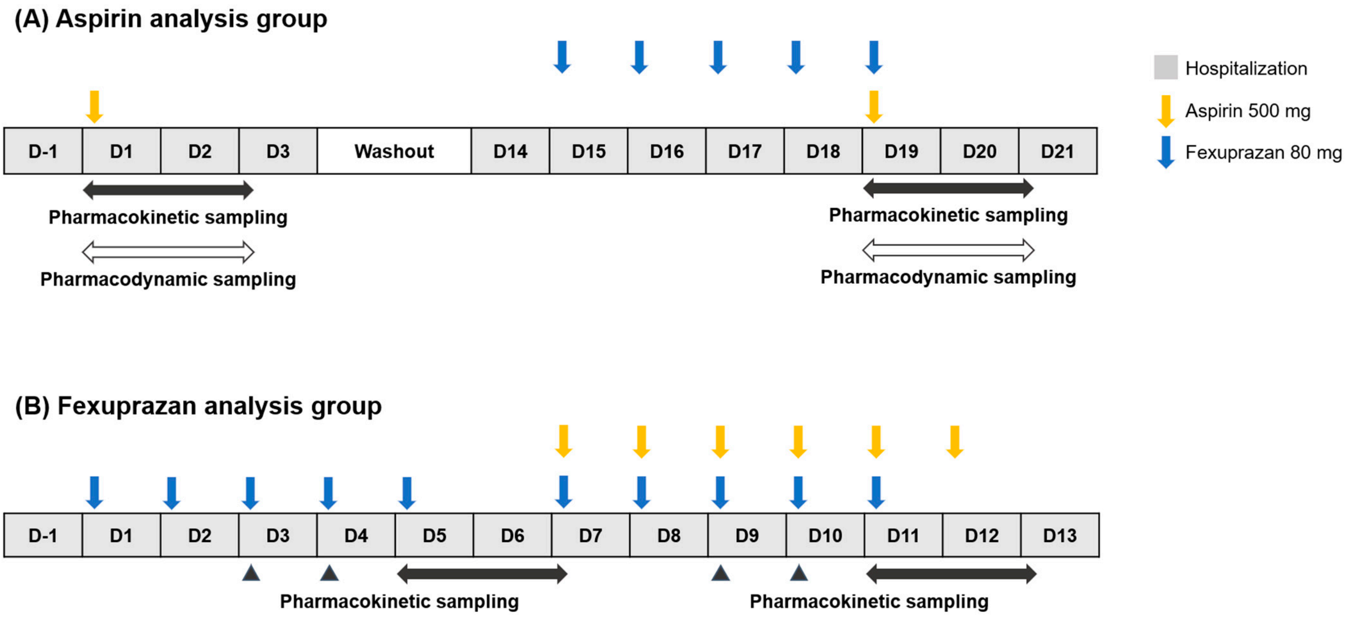

Supplementary Figure S1. Study design.

Supplementary Table S1. Analysis of variance (ANOVA) table for pharmacokinetic parameters of aspirin and fexuprazan.

Parameters	Source	df	SS	MS	F	p-value
Aspirin						
AUC_{last}	Treatment	1	0.0203	0.0203	3.38	0.1157
	Group	4	0.7364	0.1841	3.56	0.0812
	Treatment*Group	4	0.1667	0.0417	6.95	0.0194
	Subject	6	0.3107	0.0518	8.64	0.0095
	Error	6	0.0360	0.0060	.	.
AUC_{inf}	Treatment	1	0.0202	0.0202	3.28	0.1199
	Group	4	0.7358	0.1840	3.59	0.0799
	Treatment*Group	4	0.1645	0.0411	6.69	0.0212
	Subject	6	0.3078	0.0513	8.34	0.0104
	Error	6	0.0369	0.0062	.	.
C_{max}	Treatment	1	0.2974	0.2974	2.22	0.1668
	Group	4	1.9142	0.4786	2.15	0.1917
	Subject	6	1.3339	0.2223	1.66	0.2276
	Error	10	1.3372	0.1337	.	.
Fexuprazan						
$AUC_{\tau,ss}$	Treatment	1	0.2801	0.2801	44.31	<0.001
	Group	5	2.5811	0.5162	1.27	0.4012
	Subject	5	2.0394	0.4079	64.53	<0.001
	Error	10	0.0632	0.0063	.	.
$C_{max,ss}$	Treatment	1	0.0704	0.0704	5.09	0.0477
	Group	5	2.5896	0.5179	1.34	0.378
	Subject	5	1.9325	0.3865	27.96	<.0001
	Error	10	0.1382	0.0138	.	.

If the fixed effect of the interaction between treatment and group was not statistically significant, analysis of variance was performed excluding the corresponding effect.
Abbreviations: df, degree of freedom; SS, sum of squares; MS, mean square; F, F-test statstic.

Supplementary Table S2. Pharmacokinetic parameters of salicylic acid, an active metabolite of aspirin, determined after a single oral administration of aspirin 500 mg alone and coadministered with fexuprazan 80 mg.

Parameters	Aspirin 500 mg + Fexuprazan 80 mg (N=11)	Aspirin 500 mg only (N=11)
AUC_{last} (h·mg/L)	209.89 ± 121.86	207.98 ± 142.16
AUC_{inf} (h·mg/L)	214.58 ± 122.31	214.25 ± 142.05
C_{max} (mg/L)	29.79 ± 6.49	27.73 ± 6.23
Metabolic ratio[a]	40.93 ± 13.18	42.52 ± 16.61

All data are presented as mean ± standard deviation.

Abbreviations: AUC_{last}, area under the concentration-time curve (AUC) from time 0 to the time of the last quantifiable concentration; AUC_{inf}, AUC from 0 to infinity; C_{max}, maximum plasma concentration.

[a] Ratio of AUC_{inf} of salicylic acid to AUC_{inf} of aspirin

Supplementary Table S3. Pharmacokinetic parameters of M14, a metabolite of fexuprazan, determined after multiple oral administrations of fexuprazan 80 mg alone and coadministered with aspirin 500 mg.

**Parameters**	**Fexuprazan 80 mg + Aspirin 500 mg (N=11)**	**Fexuprazan 80 mg only (N=11)**
$AUC_{\tau,ss}$ (h·μg/L)	3095.05 ± 749.42	3089.57 ± 690.99
$C_{max,ss}$ (μg/L)	300.55 ± 107.88	282.00 ± 96.01
Metabolic ratio[a]	4.17 ± 2.68	3.16 ± 1.73

All data are presented as mean ± standard deviation.

Abbreviations: $AUC_{\tau,ss}$, area under the concentration-time curve for a dosing interval at steady state; $C_{max,ss}$, maximum plasma concentration at steady state.

[a] Ratio of AUC of M14 to $AUC_{\tau,ss}$ of fexuprazan
